# Elucidating the Mechanism of Electro-Adsorption on Electrically Conductive Ultrafiltration Membranes via Modified Poisson-Boltzmann Equation

**DOI:** 10.3390/membranes14080175

**Published:** 2024-08-10

**Authors:** Muhammad Usman, Shahrokh Vahedi, Sarah Glass, Volkan Filiz, Mathias Ernst

**Affiliations:** 1 Institute of Water Resources and Water Supply, Hamburg University of Technology, Am Schwarzenberg-Campus 3, 21073 Hamburg, Germany; shahrokh.vahedi@tuhh.de (S.V.); mathias.ernst@tuhh.de (M.E.); 2 Institute of Membrane Research, Helmholtz-Zentrum Hereon, Max-Planck-Straße 1, 21502 Geesthacht, Germany; sarah.glass@hereon.de (S.G.); volkan.filiz@hereon.de (V.F.)

**Keywords:** electrofiltration, dead-end ultrafiltration, adsorption, electrostatic force, water treatment

## Abstract

Electrically conductive membranes (ECMs) were prepared by coating porous ethylenediamine-modified polyacrylonitrile (PAN-EDA) UF membranes with an ultrathin layer of platinum (Pt) nanoparticles through magnetron sputtering. These ECMs were used in electrofiltration to study the removal of brilliant blue dye from an aqueous solution under positive electrical potentials (0–2.5 V). Negative electrical potentials (−1.0–−2.5 V) were also investigated to regenerate the membrane by desorbing the dye from the ECM surface. At +0 V, the EC PAN-EDA membrane adsorbed the dye due to its intrinsic positive charge. Application of −2.0 V resulted in a maximum of 39% desorption of the dye. A modified Poisson-Boltzmann (MPB) model showed that −2.0 V created a repulsive force within the first 24 nm of the membrane matrix, which had a minimal effect on dye ions adsorbed deeper within the membrane, thus limiting the electro-desorption efficiency to 39%. Moreover, increasing positive potentials from +0.5 V to +2.5 V led to increased dye electro-adsorption by 9.5 times, from 132 mg/m^2^ to 1112 mg/m^2^ at pH 8 (equivalent to the membrane’s isoelectric point). The MBP simulations demonstrated that increasing electro-adsorption loadings are related to increasing attractive force, indicating electro-adsorption induced by attractive force is the dominant mechanism and the role of other mechanisms (e.g., electrochemical oxidation) is excluded. At pH 5, electro-adsorption further increased to 1390 mg/m^2^, likely due to the additional positive charge of the membrane (zeta potential = 9.2 mV) compared to pH 8. At pH 8, complete desorption of the dye from the ECM surface was achieved with a significant repulsive force at −2.0 V. However, as pH decreased from 8 to 5, the desorption efficiency decreased by 3.9% due to the membrane’s positive charge. These findings help elucidate the mechanisms of electro-adsorption and desorption on ECMs using dye as a model for organic compounds like humic acids.

## 1. Introduction

Ultrafiltration (UF) is recognized as a high-efficiency membrane separation technology and finds its applications in both the production of clean, potable water and wastewater treatment [1,2]. UF membranes are widely used for removing a broad spectrum of pathogens (e.g., bacteria, viruses) and to improve water quality by filtering out suspended solids, colloidal particles, and macromolecular species [3,4]. Furthermore, UF provides consistent and reliable performance and offers energy-efficient and scalable solutions worldwide, including in developing countries [5,6]. However, it is not capable of either removing or concentrating dissolved organic water constituents such as humic substances or smaller molecules [7,8]. The conventional dead-end UF systems are predominantly not suitable to encounter the intensifying demand of removing and concentrating dissolved organic water constituents (with a molecular weight of <1000 Da) from feed water, as these constituents are 2–3 orders of magnitude smaller than the membrane pore size [9,10]. Most organic water constituents can be effectively rejected by NF membranes. The molecular weight cut-off (MWCO) of NF membranes ranges between 200 and 1000 Da [10].

In recent years, attempts have been made to expand the selectivity of UF via the application of electrically conducting membranes (ECMs) [11,12,13]. ECMs have shown promising results, with a marginal additional energy requirement (up to 30 kW per m^3^ of treated water) arising from the applied potential compared to the energy required for UF operation [14]. ECMs are synthesized through the deposition of nanolayers of metal (e.g., Au, Pt) nanoparticles as well as through the deposition and cross-linking of conductive materials such as carbon nanotube (CNT) networks on a porous UF membrane. ECMs have been demonstrated to efficiently remove natural organic matter (NOM) such as humic substances, small dye molecules, and heavy metal ions by applying small voltages to their surface [14,15]. In addition, the ECMs have been applied for long-term recycling, not only for the removal of NOM without losing electro-adsorption capacity but also to concentrate charged dissolved organic substances [15].

When an electrical potential is applied to the ECM surface, various processes may occur at the interface between the membrane and water, including electrochemical oxidation reactions, local pH changes through water electrolysis, and electrostatic interactions [12,16,17,18,19,20,21]. Electrochemical oxidation involves the generation of hydroxyl radicals (OH^•^), hydrogen peroxide, and reactive oxygen species [22,23,24]. Local pH changes along the membrane surface result from water electrolysis [12]. Electrostatic interactions, which depend on the applied potential, contribute to electro-adsorption and desorption [14,15,18,25], electrophoresis, and electroosmosis [15,26]. These mechanisms are known to enhance the selectivity of the membrane [12,27,28,29]. Among these mechanisms, electrostatic forces (i.e., electrostatic repulsion and attraction) are considered the primary mechanisms between the target compound and the membrane surface [19]. However, there is a lack of reported work utilizing a theoretical model based on the modified Poisson–Boltzmann (MPB) equation to explain and model the mechanisms of electrostatic attraction leading to electro-adsorption and electrostatic repulsion leading to electro-desorption.

This study aims to investigate the electro-adsorption and desorption processes of a negatively charged dye molecule on an EC UF membrane using a theoretical model. Dead-end electrofiltration experiments were conducted at varying positive and negative electrical potentials. Brilliant blue (BB) dye served as the negatively charged organic molecule stable over the whole pH range. A theoretical model based on the MPB equation was applied to calculate the potential distribution and concentration profiles of BB ions as a function of distance and applied electrical potential from the EC membrane surface. The model assessed the electrostatic forces exerted on the BB ions under the influence of an applied external potential on the membrane surface. The UF membrane is made of polyacrylonitrile (PAN), surface-functionalized with ethylenediamine (EDA), and made EC by depositing a porous nanolayer of Pt through magnetron sputtering. In this work, we also demonstrate how the dye adsorbs the intrinsic positive charge of the membrane and can be electrically desorbed from the membrane through the process of electrostatic repulsion. The impact of different pH conditions on intrinsic adsorption, electro-adsorption, and electro-sorption was evaluated as well.

## 2. Material and Methods

### 2.1. Electrically Conductive Ultrafiltration Membrane

Virgin polyacrylonitrile (PAN) UF membrane surface-functionalized with ethylenediamine (EDA) was used as a porous active material for establishing the EC UF membrane. The synthesis and surface modification of PAN with EDA, referred to as PAN-EDA hereafter, was discussed in our previous works [30,31]. The membrane is asymmetric and includes a non-woven polyphenylene sulfide (PPS) support layer to provide mechanical integrity to the thin active layer. The membrane total thickness is 200 µm, with each active and support layer being 100 µm thick. The size of the membrane was 10 cm x 5 cm. In these studies, the PAN-EDA was characterized to confirm the successful modification. The PAN-EDA membrane possessed amidine and amine groups in its membrane structure. The isoelectric point (pH_IEP_) of the membrane lies at 7.8 ± 0.2. The pore size and average surface porosity of the PAN-EDA membrane were 12.2 ± 6.1 nm and 9.3 ± 0.6%, respectively. The hydrophilicity, measured by water contact angle, of the membrane was 41.6 ± 1.3°. The membrane exhibited a relatively large pure water permeability (PWP) of 1770 ± 122 L·m^2^·h^−1^·bar^−1^ [15,30]. The MWCO of the membrane was estimated to be 85 kDa, calculated using Equation (1) documented elsewhere [32]. In this equation, $r_{p}$ is the mean pore radius.

(1) $$MWCO\;(Da)={\left(\frac{r_{p}}{0.33}\right)}^{1/0.46}$$

In our recent study [15], ECM was established by magnetron sputtering of Pt nanoparticles to form a Pt nanolayer of 20 nm using Sputter Coater (SCD 005, Baltec Inc., Balzers, Lichtenstein). After Pt sputtering, scanning electron microscopy (SEM, Merlin SEM, Zeiss, Jena, Germany) investigations had shown that porous nanolayers consisting of Pt nanoparticles were successfully formed on both sides of the membrane (Figure S3B, Supplementary Materials). The Pt nanoparticles (NPs) are concentrated solely on the membrane surface, not entering the deeper matrix. At the membrane surface, interconnected cracks appeared in the porous Pt nanolayer (Figure S3C), and these cracks allowed the water to flow through the Pt-sputtered membranes.

The obtained membrane exhibited an electrical conductivity of 2.42 × 10^6^ S/m, which was determined using a two-point method described elsewhere [33]. The PWP of the EC PAN-EDA was 1299 ± 101 L·m^2^·h^−1^·bar^−1^ and the hydrophilicity was 43.9° ± 2.2 [15], and it was still larger than the widely used PES membrane [11]. In the current study, we have used the same sputtering technique to establish EC PAN-EDA membranes and to use them for electrofiltration experiments.

The active side of the membrane contacting the feed functioned as the working electrode, while the support side contacting the permeate acted as the counter electrode. Positive cell potentials were applied to the membrane working electrode to facilitate electrostatic attraction between the membrane surface and ionic dye molecules for electro-adsorption. For membrane regeneration via the electro-desorption process, where electrostatic repulsion between the similarly charged membrane surface and ionic dye molecules is crucial, the cell polarity was reversed to negative at the membrane working electrode.

### 2.2. Feed Solution

**Figure 1:**
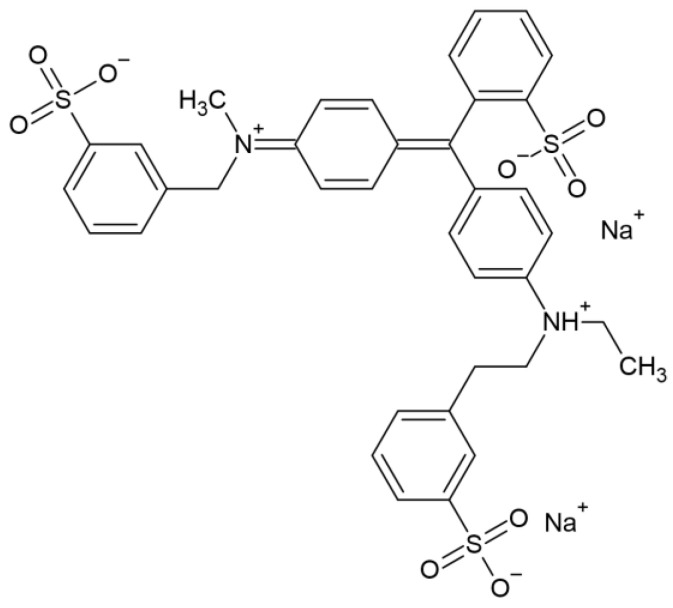
Molecular structure of brilliant blue (BB) dye.

**Figure A1:**
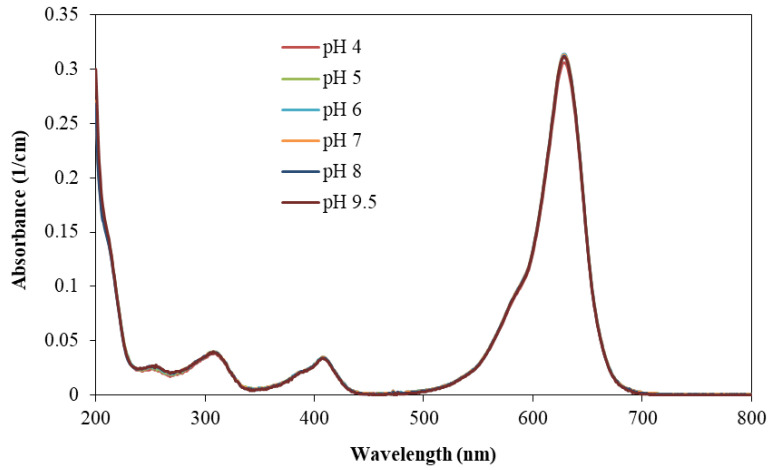
Absorption spectra of an aqueous solution containing 2.5 mg/L BB dye with an ionic strength of 1 mM at different pHs. Absorption spectra reveal an insignificant effect of solution pH on maximum light absorption, which appears at 629 nm.

Brilliant blue dye (BB dye, molecular weight = 792.8 g/mol, CAS-number: 3844-45-9) was chosen as a model organic water constituent for this study. BB dye exhibits a negative charge, with a zeta potential of approximately −2 mV, in aqueous solutions at pH 5 and 6 [34]. Additionally, this dye acts as a monovalent anion in the pH range of 7 to 9 [35]. The findings of these studies illustrate that the zeta potential of the BB dye remains negative within the studied pH range of 5–8. Moreover, the dye exhibits similar absorption spectra at various pH levels (Figure A1). BB dye was obtained from Merck Sigma-Aldrich (Darmstadt, Germany). The chemical structure of dye is shown in Figure 1.

A stock solution with a concentration of 500 mg/L was prepared. Fresh working solutions were then created by diluting the stock solution to a concentration of 2.5 mg/L and adjusting the pH to various levels (5, 6, 7, and 8) using precise additions of 0.1 M NaOH or HCl. To augment the ionic strength of the single dye feed solution, 1 mmol/L NaCl was added. The dye concentration of both feed and permeate solutions was measured photometrically at a wavelength of 629 nm.

### 2.3. Dead-End Electrofiltration Experiments

**Figure 2:**
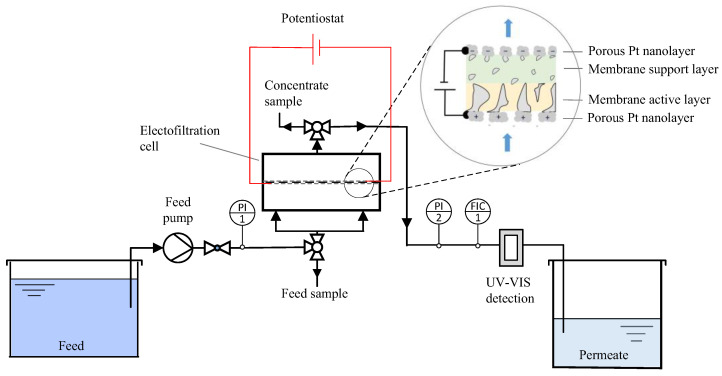
Schematic diagram shows the experimental set-up of dead-end electrofiltration used in this work. The inset illustrates the orientation of the ECM within an electrofiltration cell. The thicknesses of each membrane layer and membrane pores are not scaled.

The electrofiltration experiments were conducted using a flat sheet membrane filtration cell (CF042) fabricated by Sterlich (Kent, WA, USA), featuring an active membrane surface of 42 cm^2^ (see Figure 2). Following membrane placement, the cell was assembled using nut and bolt connections at each of its four corners. Electrical connectivity to the EC membrane was established using thin strips of titanium foil, which were subsequently linked to the potentiostat (IPS Elektroniklabor GmbH, Münster, Germany). For pumping the single dye feed solution at a constant permeate flux of 100 L·m^−2^·h^−1^, a magnetic coupled gear pump (Bronkhorst Deutschland Nord GmbH, Kamen, Germany) was used. The applied pressure to maintain a permeate flux of 100 L·m^−2^·h^−1^ was 0.11 ± 0.01 bar. Prior to the electrofiltration tests, water was filtered through the membranes at 1 bar transmembrane pressure for 1 h to prevent swelling during the investigations. The outlet of the electrofiltration cell was attached to a UV/VIS spectrophotometer (DR6000, Hach Lange GmbH, Düsseldorf, Germany) to measure the ultraviolet absorbance at 629 nm (UV_629_) using a 1 cm flow-through cuvette every 10 sec and then directed to the permeate container. Pressure sensors (Bronkhorst Deutschland Nord GmbH, Kamen, Germany) recorded the pressure at the inlet and outlet of the electrofiltration cell every 10 seconds. Permeate and dye concentrate were collected in separate containers.

In electrofiltration experiments, the EC membrane was initially tested for intrinsic adsorption without applying an external electrical potential (Table 1). This was followed by membrane regeneration through the electro-desorption process using a negative potential of −2.0 V. These experiments allowed us to investigate the dye removal by intrinsic adsorption as well as the membrane regeneration using external cell potential without the addition of additional chemicals.

In the next set of experiments, the regenerated EC membrane was first used for intrinsic adsorption, then for electro-adsorption using a positive potential, and finally, membrane regeneration was performed using a negative potential. These experiments enabled us to study the dye intrinsic adsorption and electro-adsorption at different positive cell potentials, ranging from +1.0 V to +2.5 V separately. The same methodology was adopted to investigate the effect of feed solution pH on intrinsic adsorption, electro-adsorption, and membrane regeneration through electro-desorption. In all electrofiltration tests, the cell potential was maintained at a constant value, and the current was monitored with a potentiostat.

Through the electro-desorption of intrinsically adsorbed dye, the resulting concentration exceeds the analytical limits of the spectrophotometer. Accordingly, desorption samples were collected every 2 min using the concentrate sampling port (Figure 2), diluted, and then analyzed for UV_629_ absorbance using a photometer. The dye concentration in the integrated permeate and concentrate samples was determined by comparing the UV_629_ absorbance with a calibration curve previously measured.

The adsorption loading ($q_{a}$, mg/m^2^) was calculated using the following material balance (Equation (2)) of an adsorption system:

(2) $$q_{a}=\frac{\left(C_{f}-C_{p}\right)\times V_{p}}{A_{am}}$$

where $C_{f}$ (mg/L) and $C_{p}$ (mg/L) denote the dye concentration in feed solution and integrated permeate, respectively; *V_p_* (L) is the volume of the integrated permeate; $A_{am}$ (m^−2^) is the active membrane area. When intrinsic adsorption is followed by electro-adsorption, $q_{a}$ reflects the combined adsorption loading.

The electro-desorption efficiency of the EC PAN-EDA membrane achieved through the application of negative potential (membrane active side as working electrode) was calculated using the following Equation (3)

(3) $$\mathrm{Electro}-\mathrm{desorption}\;\mathrm{efficiency}=\frac{q_{d}}{q_{a}}$$

where $q_{d}$ (mg/m^2^) denotes the electro-desorption loading achieved by applying a negative potential at the membrane working electrode.

**Table 1:** Parameters of dead-end electrofiltration experiments.

Tested Parameters	Cell Potential (V)	pH of Feed Solution	Ionic Strength (mmol·L^−1^)
Membrane intrinsic surface charge		5, 6, 7, 8	1
External positive cell potential	+1.0, +1.5, +2.0, +2.5	5, 6, 7, 8	1
External negative cell potential	−1.0, −1.5, −2.0, −2.5	5, 6, 7, 8	1

We have demonstrated in previous work that Pt and organic water constituents did not undergo electrochemical oxidation at a cell potential of 2.5 V [15], which corresponds to 1.2 V vs. Ag/AgCl reference [36,37]. Moreover, no water electrolysis was observed on the EC membrane surface until 1.2 V vs. Ag/AgCl (Figure S1, Supplementary Materials), which is equivalent to a cell potential of 2.5 V, as verified by the voltage–current density curve (Figure S2, Supplementary Materials). Accordingly, a maximum (positive and negative) cell potential of (+/−) 2.5 V was applied in this work to avoid electrochemical reactions and water splitting, which may occur at cell potentials exceeding 2.5 V [19,36].

### 2.4. Modelling Ion Concentration and Electrostatic Force

To model the ion concentration along a charged membrane surface and the electrostatic force on a particle during electrofiltration, it is crucial to determine the potential profile generated by the electric potential applied to the membrane. Traditionally, the standard Poisson–Boltzmann (SPB) model has been used to describe the electric potential profile away from the surface of an electrode or charged surface. However, the SPB model does not account for the finite volume of ions and neglects ion–ion interactions and steric effects [38,39], leading to an unrealistic prediction that allows an infinite number of counterions in the Stern layer and overestimating the potential drop with increasing distance from the charged ECM surface.

A more accurate modification, known as the Modified Poisson–Boltzmann (MPB) model, incorporates the finite volume of ions when calculating the number of counterions attracted to an oppositely charged surface. Consequently, the MPB model predicts a region along the charged (ECM) surface that is more than one molecule thick, containing only counterions. This layer is significantly thicker than the single layer of counterions predicted by the SPB model. The MPB model may thus provide a more accurate representation of electrostatic interactions between charged ECM surfaces and dye ions in electrofiltration membrane units. Equation (4) illustrates the MPB, which is used in conjunction with Equation (5) to numerically simulate the potential distribution [20]:

(4) $$\frac{d^{2}\varphi }{dx^{2}}=-\frac{eN_{a}}{\varepsilon }\frac{\sum_{i}z_{i}c_{i}^{\infty }\exp\left(-\frac{z_{i}e\varphi }{k_{b}T}\right)}{1+\sum_{i}\frac{c_{i}^{\infty }}{c_{i}^{max}}\left[\exp\left(-\frac{z_{i}e\varphi }{k_{b}T}\right)-1\right]}$$

(5) $$c_{i}^{max}=\frac{\rho }{\frac{4}{3}\pi \;R_{i}^{3}N_{A}}$$

In these equations, e denotes the elementary charge, ε  represents the permittivity of the solution (ε = 40$\varepsilon_{0}$ [20], where $\varepsilon_{0}$ denotes the permittivity of the vacuum), $N_{a}$ stands for Avogadro’s number, $k_{b}$ stands for Boltzmann constant, and *T* denotes the absolute temperature (=298 K). Additionally, $z_{i}\;$ denotes the valence of ions, $c_{i}$ denotes the bulk concentration of ions, $c_{i}^{max}$ denotes the maximum ion concentrations in a given space given ionic steric effects, ρ denotes ion the packing density (0.64 for dense random packing [20,40]), $R_{i}$ denotes the ionic radius, φ denotes the electrical potential, and x represents the distance measured from the surface. The values of key parameters are listed in Table 2 for this specific system, which comprises a binary mixture of Na^+^ and BB^−^ (negatively charged BB dye).

To solve Equation (4), boundary conditions must be established. At the surface of the membrane (x = 0), the surface potential is equal to the applied electrical potential ($\varphi_{0}$). Moreover, at an infinite distance (x = ∞) from the membrane surface, where the influence of the applied potential dissipates, the potential is zero. Based on electrochemical impedance spectroscopy, the depth within the porous membrane at which the potential reaches zero is 0.01 times the membrane thickness [32]. Accordingly, this distance was set to L = 2 µm.

After obtaining the electrical potential profiles away from the ECM surface, Equation (6) was employed to compute the concentration of individual ions as a function of distance from the membrane surface [20].

(6) $$c_{i}(x)=\frac{c_{i}^{\infty }\exp\left(-\frac{z_{i}e\varphi }{k_{b}T}\right)}{1+\sum_{i}\frac{c_{i}^{\infty }}{c_{i}^{max}}\left[\exp\left(-\frac{z_{i}e\varphi }{k_{b}T}\right)-1\right]}$$

Subsequently, the free energy between two parallel plates was evaluated using the Gibbs adsorption isotherm. This process involves coupling constant integration, where the ion concentration between the plates is incrementally changed from zero to match the excess ion concentration induced by surface potentials (as described in Equation (7)), where L denotes the plate separation. The final free energy is calculated by subtracting the free energy at an infinite plate separation from the value between the plates at specified distances, as outlined in Equation (8).

(7) $$F\left(c\right)=-k_{b}TN_{a}\int_{0}^{c}\int_{0}^{L}\left(\frac{\sum_{i}c_{i}(x)}{c_{i}^{\infty }}-1\right)dxdc$$

(8) $$F^{e}=F\left(c\right)-F(c^{\infty })$$

(9) $$F_{es}=2\pi \int_{0}^{a}\left[\frac{\partial F^{e}}{\partial x}\left(x+a+a\sqrt{1-{\left(r/a\right)}^{2}}\right)-\frac{\partial F^{e}}{\partial x}\left(x+a-a\sqrt{1-{\left(r/a\right)}^{2}}\right)\right]rdr$$

The electrostatic force ($F_{es}$) acting on a particle was calculated using the surface elementary integration method, as described in Equation (9). In this method, $\partial F^{e}/\partial x\;$ represents the derivative of the free energy function at a separation distance x, where a is a constant and equals the radius of the BB dye (=$R_{BB}$), and r is the variable radius over which integration is conducted. This approach allows for the transformation of the initial parallel plate assumption into the required and specific particle–plate interaction [20].

## 3. Results and Discussion

### 3.1. Intrinsic Adsorption and Membrane Regeneration Using External Potential

#### 3.1.1. Dye Intrinsic Adsorption

**Figure 3:**
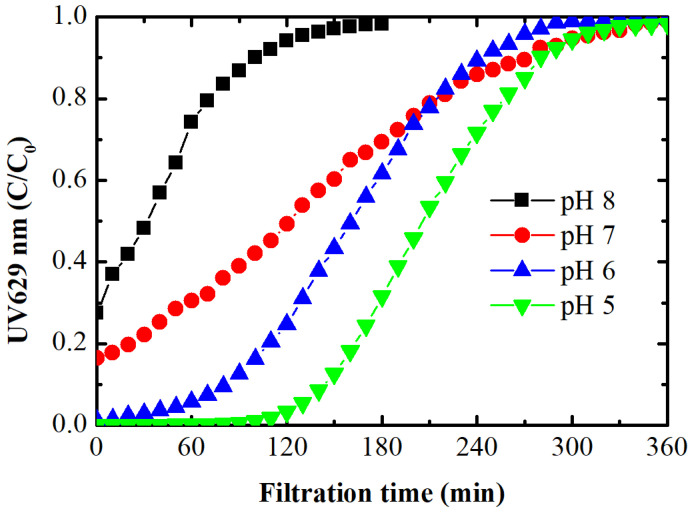
UV_629_ absorbance curves depicting intrinsic adsorption at different feed water pHs. Feed solution contains 2.5 mg/L of BB dye at an ionic strength of 1 mM.

Dead-end filtration of the dye feed solution without applied potential at different pH values is shown in Figure 3. The EC PAN-EDA membrane exhibited dye adsorption, as indicated by normalized UV_629_. The membrane is positively charged at pH < 7.8 ± 0.2, and intrinsic membrane charge increases with decreasing pH due to the protonation of amine groups (-NH_2_) in EDA to cationic form (-NH_3_^+^) to a greater extent under slight acid conditions [41]. The recorded zeta potential of the membrane was 3.7 mV ± 2.5 at pH 7, 5.2 mV ± 1.8 at pH 6, and 9.2 mV ± 2.6 at pH 5 [15]. Hence, this removal was mainly due to the electrostatic attraction of negatively charged dye to the positively charged amine, which is present in the EC PAN-EDA membrane structure. This dye rejection by size exclusion is improbable because the size of the used dye falls within the range of 1 to 2 nm, which is nearly one order of magnitude smaller than the pore size of the membrane used. In our studies, analogous adsorption mechanisms were observed for NOM and heavy metal anions like arsenate (AsO_4_^3−^) and chromate (CrO_4_^2−^) [30,31].

As the pH increased, intrinsic adsorption decreased, consistent with the zeta potential measurements. It is interesting to note that the EC PAN-EDA membrane exhibited dye adsorption at a pH corresponding to its pH_IEP_. These results suggest that some positively charged groups may still be present within the membrane structure, likely deeper within the membrane matrix, and were not accounted for in the zeta potential as here only surface measurements were considered.

**Figure 4:**
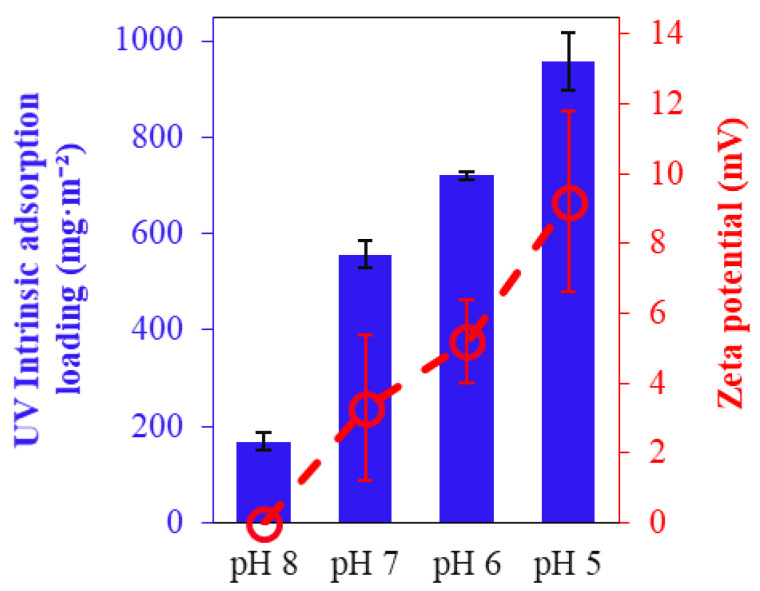
Intrinsic BB dye adsorption loadings of the EC PAN-EDA membrane at different pH values. Error bars reflect experimental uncertainty and denote the standard deviation of repeated measurements (*n* = 2) in each instance.

The calculated adsorption loadings are consistent with the zeta potential values observed at different pH levels (Figure 4). At lower pH values, where the zeta potential is more positive, the membrane exhibited higher intrinsic adsorption loadings, indicating stronger electrostatic attraction between the positively charged membrane surface and the negatively charged dye molecules. Conversely, at higher pH levels, the zeta potential decreases, leading to reduced adsorption loadings and confirming the role of electrostatic interactions in the adsorption process.

#### 3.1.2. Membrane Regeneration Using External Potential

**Figure 5:**
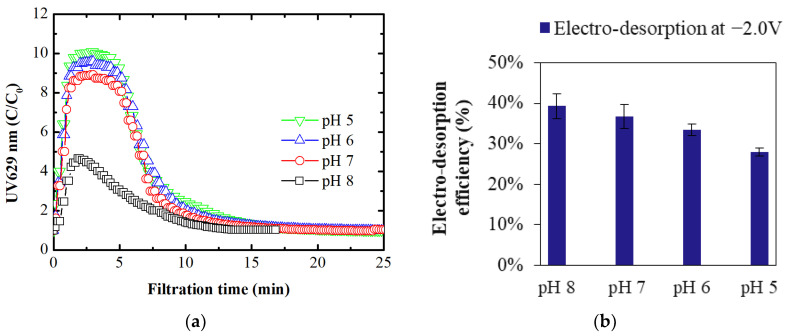
(**a**) UV absorbance curves depicting electro-desorption; (**b**) electro-desorption efficiency at different feed water pHs. A feed solution containing 2.5 mg/L BB dye at an ionic strength of 1 mM was used for electro-desorption.

The electrofiltration experiments for in situ membrane regeneration were conducted by applying a negative electrical potential to the membrane working electrode (membrane electrode as cathode). The aim was to evaluate the electro-desorption process for intrinsically adsorbed organic molecules. The same feed solution used for intrinsic and electro-adsorption experiments (2.5 mg/L BB dye) was pumped during electro-desorption experiments. The obtained UV_629_ absorbance curves depicting electro-desorption are shown in Figure 5a.

The sharp increase in UV_629_ absorbance indicates a significant release of negatively charged BB dye from the membrane surface, with electro-desorption peaking within the first 2 minutes. The highest peak occurred at pH 5, and the lowest at pH 8. At pH 5, more organic ions were adsorbed due to intrinsic positive recharge, resulting in a larger release when a negative electrical potential was applied. Conversely, at pH 8, adsorption and subsequent release were lower. After the peaks, electro-desorption tapered off for all pH levels, likely due to decreasing concentration differences between solid-phase and liquid-phase dye concentrations, slowing the transport of organic ions from the membrane structure. Nearly 90% of electro-desorption occurred within the first 10 min with the process completing in 15–20 min, depending on the desorbable load.

The results on electro-desorption efficiency (Figure 5b) indicate that the electro-desorption efficiency of the membrane at −2.0 V and pH 8 is 39%, which decreased to 37% at pH 7, 33% at pH 6, and 29% at pH 5. This decrease in efficiency can be explained by the increasing positive zeta potential of the membrane with decreasing pH. As the pH decreases, the membrane’s intrinsic positive charge increases, which likely reduces the effectiveness of the applied negative electrical potential. The stronger intrinsic positive charge of the membrane counteracts the repulsive interaction generated by the negative potential, thereby diminishing the overall electro-desorption efficiency.

**Figure 6:**
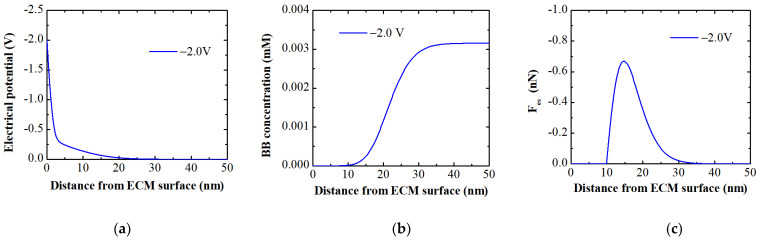
MPB model simulations; (**a**) potential profile; (**b**) concentration of BB ions; and (**c**) electrostatic force acting on BB ions as a function of distance from the ECM surface in a BB solution containing 2.5 mg/L BB dye at an ionic strength of 1 mM.

The MPB model was employed to elucidate the electro-desorption process of intrinsically adsorbed BB dye. Model simulations at −2.0 V indicated a rapid drop in electrical potential within the porous membrane depth, diminishing at approximately 23.5 nm (Figure 6a). These model simulations are consistent with previous experimental results reported by Jing et al. [32]. This drastic potential drop with depth into the porous membrane active layer can be attributed to membrane pores, specific solution resistance, and applied external potentials [19,32].

**Figure 7:**
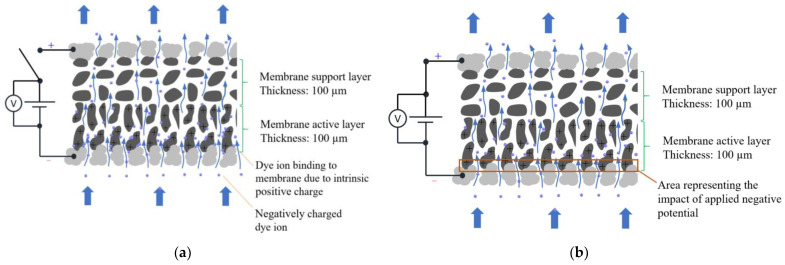
Scheme illustrating (**a**) the binding of BB dye ions to the membrane through intrinsic positive charge and (**b**) the detachment of BB dye ions from the first few nanometers of the membrane surface.

The model predicted, as expected, that applying a negative electrical potential to the membrane surface (with the membrane electrode as the cathode) would reduce the concentration of like-charged dye to zero, indicating complete desorption. Beyond this region (~11.3 nm), the concentration of counterions gradually approached the bulk concentration (Figure 6b). The modeling results also revealed the presence of an electrostatic repulsive force when potentials were applied, peaking at ~14.0 nm above the membrane surface due to steric effects. Steric effects arise from the spatial arrangement of atoms within molecules or ions, causing nonbonding interactions (e.g., physical hindrance or repulsion) between atoms when they come into close proximity. This can lead to changes in molecular shape, conformation, or reactivity [38,39]. This electrostatic repulsive force, effectively acting as a desorption force, can only detach dye within the initial few nanometers of the membrane surface. However, it does not detach organic ions that are adsorbed deep within the membrane structure (Figure 7), leading to lower dye desorption efficiency.

### 3.2. Electro-Adsorption at Varying Potential and pH

#### 3.2.1. Electro-Adsorption at Varying Positive Potential

**Figure 8:**
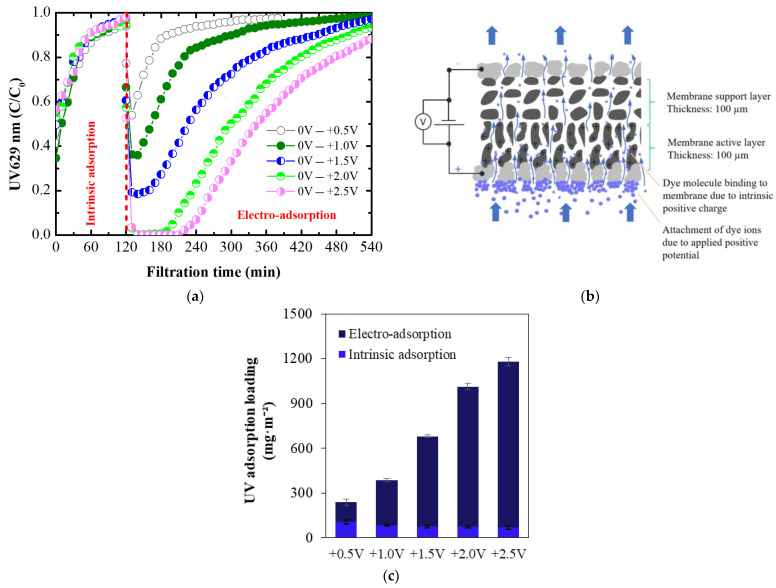
(**a**) Intrinisc adsorption curves followed by electro-adsorption; (**b**) Scheme illustrating the binding of BB dye ions to the membrane through intrinsic positive charge and applied positive potential; and (**c**) UV intrinsic and electro-adsorption loadings with varying electrical potential at pH 8.

The electro-adsorption of dye on ECMs was investigated at varying potentials ranging from +0.5 V to +2.5 V and at pH 8. The dye adsorbed to the membrane structure due to its intrinsic charge. Therefore, prior to investigating electro-adsorption at these positive potentials, the adsorption caused by the intrinsic membrane charge was studied (Figure 8a). Upon reaching full saturation with dye, applying a positive potential to the membrane surface (with the membrane as the working electrode) resulted in an immediate decrease in the UV_629_ value, indicating that more dye has adsorbed onto the membrane’s surface through the additional impact of the positive electrical potential. These results indicate that the binding sites for adsorption due to the intrinsic membrane charge and electro-adsorption induced by external positive potential are different. Adsorption, due to the intrinsic membrane charge, occurs at specific binding sites within the membrane structure. In contrast, electro-adsorption induced by external positive potential may cause dye binding via electrostatic attraction directly to the membrane surface through electrostatic attractive forces, as depicted in Figure 8b. The results in Figure 8a further indicate that increasing the positive potential not only improved BB adsorption but also extended the duration of lower dye concentrations in the permeate. At +2.0 V and +2.5 V, the complete removal of dye was achieved. This suggests that higher positive potentials enhance the efficiency of dye removal by increasing the electrostatic attraction between the dye ion and the membrane surface, resulting in more effective and prolonged electro-adsorption.

The intrinsic adsorption and electro-adsorption loadings of the membrane for dye were calculated and shown in Figure 8c. The intrinsic adsorption decreased slightly with an increasing filtration cycle. As the applied positive potential increased, the dye electro-adsorption loadings also increased. At +0.5 V, UV electro-adsorption loading was 132 mg/m^2^, which increased to 1112 mg/m^2^ at the highest potential of +2.5 V. The low electro-adsorption at +0.5 V is likely due to the very low current density of 0.1 A/m^2^, which is insufficient for strong electrostatic attraction between the dye ion and the membrane surface. At +1.0 V, the UV electro-adsorption loading increased by more than 127%, likely due to the increased current density of 0.2 A/m^2^. At the highest potential of +2.5 V, the current density reached 3.7 A/m^2^, further enhancing electrostatic attraction and resulting in more effective dye adsorption. These findings demonstrate the significant impact of applied potential and current density on dye electro-adsorption efficiency.

Furthermore, the ECMs demonstrated excellent electrical conductivity following electro-adsorption at +2.5 V. The used EC membranes exhibited an electrical conductivity of 1.94 ± 0.3 × 10^6^ S/m, which is slightly lower than that of the fresh EC membrane. This reduction is attributed to the binding of BB molecules to the EC membrane surface. The SEM images (Figure S3C,D, supporting information) show that the membrane morphology remains largely unchanged before and after electro-adsorption. The visual appearance of the EC membrane also remained unaltered (Figure S4, supporting information). Additionally, we used nanoparticle tracking analysis (NTA) using NanoSight LM10 (Nanosight, Malvern, UK) to examine the filtrate for potential Pt leaching. No particles were detected in the filtrate, indicating Pt nanoparticles were not leaching to a greater extent from the EC membrane surface. The electrical conductivity measurements, SEM images, and absence of Pt nanoparticles in the filtrate together provide evidence that relevant Pt leaching did not occur.

**Figure 9:**
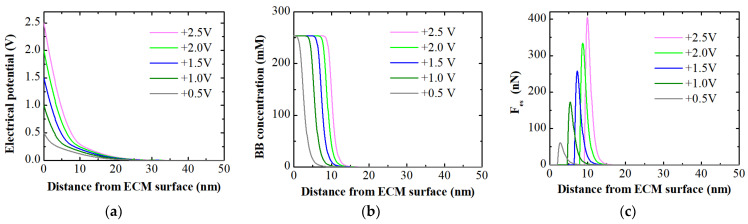
MPB model simulations for: (**a**) potential profile; (**b**) concentration of BB ions; (**c**) Electrostatic force acting on dye counterion as a function of distance from the ECM surface and applied positive potentials in a feed solution containing 2.5 mg/L BB dye at an ionic strength of 1 mM.

The MPB model was exercised to provide qualitative insights into the observed experimental results on electro-adsorption. The simulations included the electrical potential profiles, BB concentration along the ECM surface, and electrostatic attractive force as a function of applied membrane potential and distance from the membrane surface (Figure 9). As expected, as the distance from the membrane surface increases, the potential decreases for all applied positive potentials. The concentration of negatively charged dye along the membrane surface reached a maximum of approximately 255 mM. The maximum concentration is a function of the finite volume of BB ions that can accumulate or fit on the charged surface [22].

Modeling results further indicated the presence of a strong electrostatic attractive force upon application of positive potentials. The maximum force was observed at 2.5–10 nm above the surface due to steric effects [38]. Similar to experimentally calculated electro-adsorption loadings at +0.5 V (Figure 8c), the width of the counterion zone as well as the electostatic force simulated by the MPB model were relatively small compared to other positive potentials. Increasing positive potential widens the counterion zone but does not increase the maximum physically bound BB dye concentration. Beyond this zone, the concentration of counter ions gradually converges toward the bulk concentration. In contrast, both position as well as the magnitude of the force peak increased with increasing positive potential, mirroring the trend observed in electro-adsorption loadings. A maximum attractive force of 399 nN was achieved at +2.5 V. This force peak resulted from the finite volume of hydrated ions that can accumulate at the charged ECM surface, limiting the ion concentration and thereby capping the maximum attractive force. The model results highlight that while the counterion zone was influenced by positive potentials, the actual dye concentration bound to the membrane surface remained constrained by the physical properties of hydrated ions. The electrostatic force increased proportionally with positive potential, analogous to the behavior observed in experimentally determined electro-adsorption loadings. Based on these findings, it can be concluded that the electrostatic attractive force serves as the primary mechanism for binding dye to the membrane surface, while the role of other processes such as pollutant degradation via electrochemical oxidation is insignificant.

#### 3.2.2. Electro-Adsorption at Varying pH

**Figure 10:**
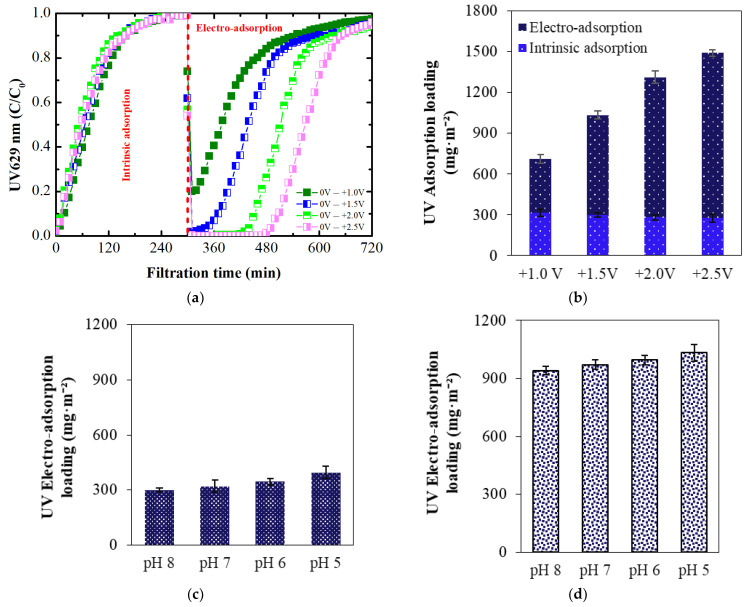
(**a**) BB dye intrinsic adsorption followed by electro-adsorption at pH 5; (**b**) UV intrinsic and electro-adsorption loadings at pH 5; and electro-adsorption loadings at (**c**) +1.0 V and (**d**) at +2.0 V.

The influence of membrane intrinsic charge on electro-adsorption at varying applied positive potentials was studied (Figure 10a).

Similar to pH 8, intrinsic adsorption decreased slightly with increasing filtration cycles (Figure 10b). Comparing electro-adsorption at pH 8 (Figure 8a) and pH 5 (Figure 10a), dye electro-adsorption rates were improved for all applied positive potentials, highlighting the positive effect of membrane intrinsic charge. For example, at +1.0 V, the lowest normalized dye concentration in the permeate was 0.21 at pH 5, compared to 0.33 at pH 8. To further elucidate this effect, electro-adsorption loadings at +1.0 V (Figure 10c) and +2.0 V (Figure 10d) were compared. These results indicate that intrinsic charge, influenced by different solution pH, has a more pronounced effect at lower positive potentials like +1.0 V compared to +2.0 V. Specifically, at +1.0 V, this effect was more pronounced at pH 5 (31% increase in electro-adsorption loading compared to pH 8) than at pH 7 (7% increase in electro-adsorption loading compared to pH 8).

The performance of the EC membranes was compared to the widely used activated carbon (AC) adsorption for the removal of organic water constituents. The calculated electro-adsorption loading of the EC membrane at +2.5 V and pH 8 was 2365 mg·g^−1^, based on the total membrane weight. This is significantly higher than the adsorption loading of AC for BB dye, which achieves a maximum of 80 mg·g^−1^ under optimal acidic conditions (pH 4) [42]. This demonstrates the superior efficiency of EC membranes for water treatment applications.

### 3.3. Membrane Regeneration Using Electro-Desorption at Varying Potential and pH

#### 3.3.1. Membrane Regeneration Using Electro-Desorption at Varying Potential

After the membrane was fully saturated through intrinsic adsorption and electro-adsorption at +2.0 V, in situ membrane regeneration was conducted by applying a negative electrical potential to the membrane, which acted as a cathode. This regeneration step aimed to evaluate how effectively the BB dye, predominantly attracted to the ECM surface by electrostatic attractive forces, could be desorbed from the membrane when different negative potentials were applied.

**Figure 11:**
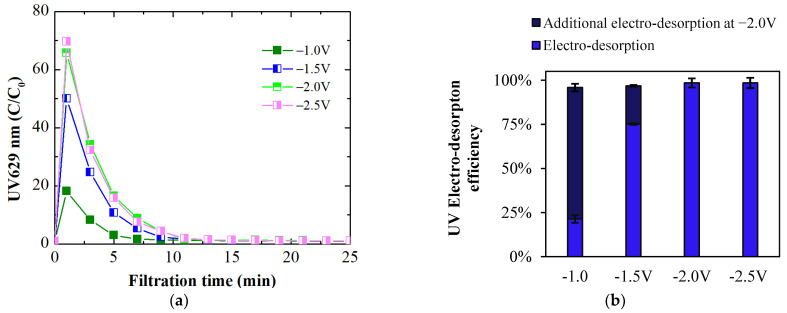

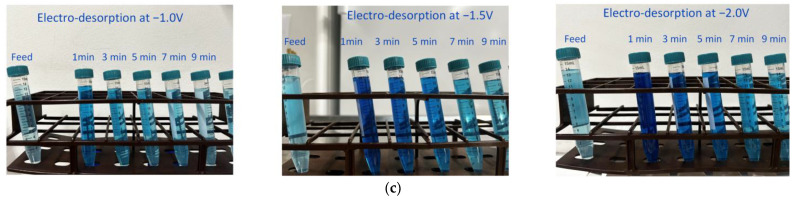
(**a**) UV electro-desorption curves; (**b**) electro-desorption loadings at pH 8 and varying negative electrical potentials. The dark blue column represents the proportion of additional electro-desorption at −2.0 V following the initial electro-desorption at a potential lower than −2.0 V; (**c**) the visual appearance of dye color in the desorbate at −1.0 V, −1.5 V, and −2.0 V at pH 8.

For these electro-desorption experiments, a feed solution containing 2.5 mg/L of BB dye at pH 8 and 1 mM ionic strength, which was the same concentration used in the initial dye adsorption experiments, was used. The UV_629_ absorbance curves illustrating the extent and rate of electro-desorption are shown in Figure 11a. These curves provide insights into desorption dynamics and the optimal conditions for membrane regeneration. At the lowest cell potential of −1.0 V, the electro-desorption peak appearing at 1 min was the lowest, resulting in an electro-desorption efficiency of 21.3% (Figure 11b). Following −1.0 V, −2.0 V was applied to desorb additional dye with stronger electrostatic repulsion (UV data presented in Figure 11b as additional electro-desorption at −2.0 V), resulting in an overall desorption efficiency of 95.8%. At −1.5 V, the electro-desorption efficiency improved to 75.1%. Subsequent additional dye desorption at −2.0 V following −1.5 V further increased the efficiency to 96.8%. Under electro-desorption conditions at −2.0 V, the C/C₀ peak reached 65.8, achieving an electro-desorption efficiency of 98.5% (Figure 11b). At −2.5 V, the electro-desorption peak was slightly greater than at −2.0 V, but the electro-desorption efficiency remained at 98.4%. The visual appearance of the desorbate at −1.0 V, −1.5 V, and −2.0 V is depicted in Figure 11c, showing a noticeable increase in blue color intensity as the applied negative potential increases. By 9 min, the color of the desorbate closely matches that of the feed color.

The electro-desorption efficiency at −1.0 V was low at 21.3%, likely due to the inadequate current density of −0.2 A/m^2^, which hindered effective electrostatic repulsion between the dye ion and the membrane surface. Increasing the potential to −1.5 V and further to −2.0 V raised the current densities (up to −1.6 A/m^2^ at −2.0 V), significantly enhancing electrostatic repulsion. This improvement led to more efficient detachment of dye ions from the membrane surface. These results highlight the critical influence of applied negative potential and resulting current density on the electro-desorption efficiency of organic molecules, such as dyes, from membranes.

**Figure 12:**
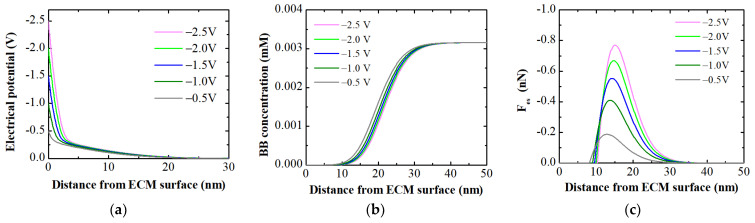
MPB model simulations: (**a**) potential profile; (**b**) concentration of BB ions; and (**c**) electrostatic force acting on BB ions as a function of distance from the ECM surface in a feed solution containing 2.5 g/L BB dye at an ionic strength of 1 mM.

Like electro-adsorption, the MPB model is used to elucidate the electro-desorption process of dye ions attracted to the membrane surface through electrostatic attraction. The model simulations (Figure 12a) revealed a significant potential drop with increasing depth into the porous membrane at all simulated negative potentials (−0.5 to −2.5 V), similar to what was predicted at positive potentials. The most critical aspect of the simulation related to electro-desorption shows an almost complete repulsion of counter dye ions (Figure 12b), indicated by a zero concentration in the vicinity (up to 11.2 nm, depending on the applied potential) of the membrane surface. With increasing negative potential, the electrostatic repulsive force increases. The overall force maxima at −2.0 V and −2.5 V are located at 14.9 nm and 15 nm above the surface (Figure 12c), respectively, resulting in the almost complete detachment (up to 98%) of electrostatically adsorbed dye ions from the membrane surface.

These simulations explicate the superior electro-desorption efficiency (up to 98%) of electrostatically adsorbed dye ions compared to the electro-desorption efficiency (maximum of 39%, Figure 5b) of ions adsorbed by the intrinsic membrane charge at the same applied negative potential (i.e., −2.0 V). This indicates that electro-desorption effectively detaches ions from the membrane surface that are either directly attracted to the membrane surface through electro-adsorption or adsorbed via intrinsic membrane charge within the influence of electrostatic repulsion (up to ~23.5 nm from the membrane surface). In other words, the electro-desorption process is particularly effective in removing charged ions that have been electrostatically attracted to the membrane surface.

#### 3.3.2. Membrane Regeneration Using Electro-Desorption at Varying pH

**Figure 13:**
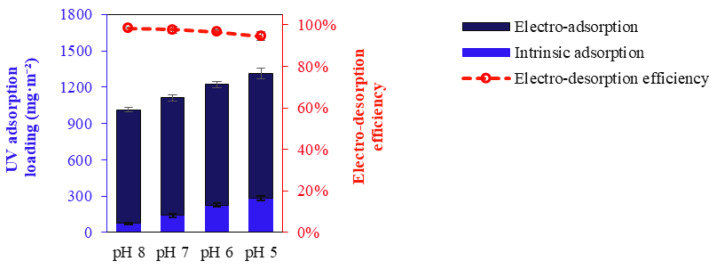
UV intrinsic adsorption, electro-adsorption (at +2.0 V), and electro-desorption efficiency (at −2.0 V) of EC PAN-EDA for dye at various feed water pH and ionic strengths of 1 mM.

Membrane regeneration at various pH levels was investigated to understand these pH-dependent interactions, which are essential for optimizing the electro-desorption process, especially in applications sensitive to pH variability. The results are presented in Figure 13.

Figure 13 demonstrates that intrinsic adsorption increases with decreasing pH, while the impact of pH on electro-adsorption at +2.0 V is minor. Electro-desorption efficiency decreases as pH drops from the pHI_EP_ to more positive membrane charges. Electro-desorption efficiency declines from 98.5% at pH 8 to 94.6% at pH 5, indicating the negative impact of positive membrane charge on electro-desorption. Optimal conditions for electro-desorption are achieved at pH levels equivalent to pH_IEP_. At these pH conditions, the membrane carries a neutral charge, facilitating a strong impact on electrostatic repulsion and thereby enhancing the efficiency of counterion detachment from the membrane surface during the regeneration process.

## 4. Conclusions

In this study, we described how applying an electrical potential to the surface of an EC PAN-EDA UF membrane can adsorb BB dye via electrostatic attraction and desorb via electrostatic repulsion. The significant outcomes of this study are:

- When no electrical potential was applied to the ECM surface, adsorption was due to the membrane’s intrinsic positive charge from amine groups. This intrinsic adsorption increased with decreasing pH due to the enhanced protonation of the amine groups.
- Applying a negative potential of −2.0 V led to partial electro-desorption (up to 39%) of BB ions via electrostatic repulsion. The MPB model indicated that this repulsive force extends up to 24 nm from the membrane surface, enabling this partial regeneration. At a constant potential, electro-desorption efficiency decreased as pH fell below the pH_IEP_ due to the membrane’s increased intrinsic positive charge, countering the negative potential’s repulsive force.
- Using the ECM as an anode and applying positive potentials resulted in electrostatic attraction, facilitating the electro-adsorption of aqueous counterions on the ECM surface. The extent of electro-adsorption increased with higher applied positive potentials. The MPB model provided a comprehensive explanation for the experimentally observed electro-adsorption results. The electrostatic attractive force predicted by the MPB model at different positive potentials qualitatively agreed with the determined electro-adsorption loadings.
- The adsorption of BB ions on the ECM involves distinct mechanisms depending on the conditions: intrinsic membrane charge facilitates binding within the structure, while external positive potential induces electro-adsorption via direct electrostatic attraction.
- Applying a negative potential to the ECM when it serves as the cathode in electrofiltration utilizes electrostatic repulsion to detach negatively charged organic ions from the ECM surface. The magnitude of this negative potential, requiring a potential of at least −2.0 V for complete desorption, plays a crucial role in ensuring the membrane’s effective regeneration and sustained performance. Optimal electro-desorption occurred at pH levels equal to pH_IEP_, where the membrane’s neutral charge maximized electrostatic repulsion, enhancing counterions detachment during membrane regeneration.

## Figures and Tables

**Table 2:** Constants and parameters used to simulate the distribution of electrical potential from the membrane surface.

Universal Constants			
e (C)		1.6 × 10^−19^	
$N_{a}$ (mol^−1^)		6.022 × 10^23^	
$\varepsilon_{0}$ (F/m)		8.85 × 10^−12^	
$k_{b}$ (J/K)		1.38 × 10^−23^	
Ions Parameters			
BB^−^		Na^+^	
$R_{BB}$ (nm)	1.0	$R_{Na}$ (nm)	0.45
$z_{BB}\;$	−1	$z_{Na}$	+1
$c_{BB}^{\infty }$ (mmol/l)	0.0032	$c_{Na}^{\infty }$ (mmol/l)	0.0032

## Data Availability

The data presented in this study are contained within the article, further inquiries can be directed to the corresponding author.

## Supplementary Materials

The following supporting information can be downloaded at: https://www.mdpi.com/article/10.3390/membranes14080175/s1, Figure S1: Cyclic voltammogram of a Pt electrode in 0.5 mol·L^−1^ H_2_SO_4_ solution at a scan rate of 50 mV·s^−1^, scanned from −0.2 V to +1.2 V vs. Ag/AgCl [37]; Figure S2: V-I curve for cell potential in feed solution (natural organic matter= 12 mg·L^−1^ with NaCl = 1 mmol·L^−1^ at pH 7 and filtration flux = 100 L·m^−2^·h^−1^ [15]; Figure S3: SEM imaging of (A) uncoated PAN-EDA membrane; (B) cross-section of EC PAN-EDA membrane; (C) EC PAN-EDA membrane: before electro-adsorption; (D) EC PAN-EDA membrane: after electro-adsorption with 50K magnification; Figure S4: Visual appearances of the EC membrane; (A) Before electro-adsorption; and (B) After membrane regeneration.
