# Supplementary material for: Elucidating the Mechanism of Electro-Adsorption on Electrically Conductive Ultrafiltration Membranes via Modified Poisson-Boltzmann Equation
Source: Membranes (Basel). 2024 Aug 10;14(8):175. doi: 10.3390/membranes14080175 (PMC11356185; doi:10.3390/membranes14080175)
Supplement: Supplementary file 1 [file membranes-14-00175-s001.zip › membranes-3128488-supplementary.pdf]

Supplementary Materials

Article

Elucidating the mechanism of electro-adsorption on electrically conductive ultrafiltration membranes via modified Poisson-Boltzmann equation

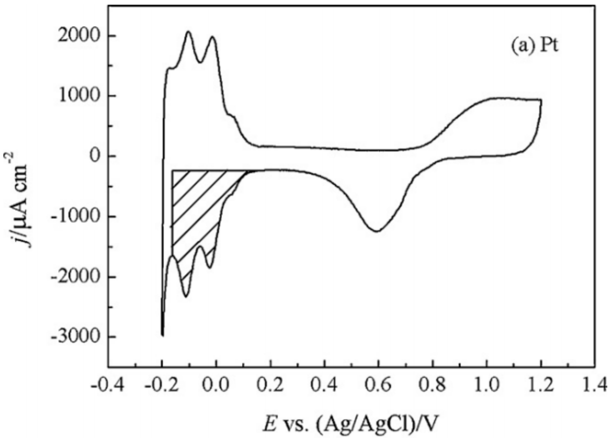

Figure 1S. Cyclic voltammogram of a Pt electrode in 0.5 mol·L^{-1} H_2SO_4 solution at a scan rate of 50 mV·s^{-1}, scanned from -0.2 V to +1.2 V vs. Ag/AgCl [1].

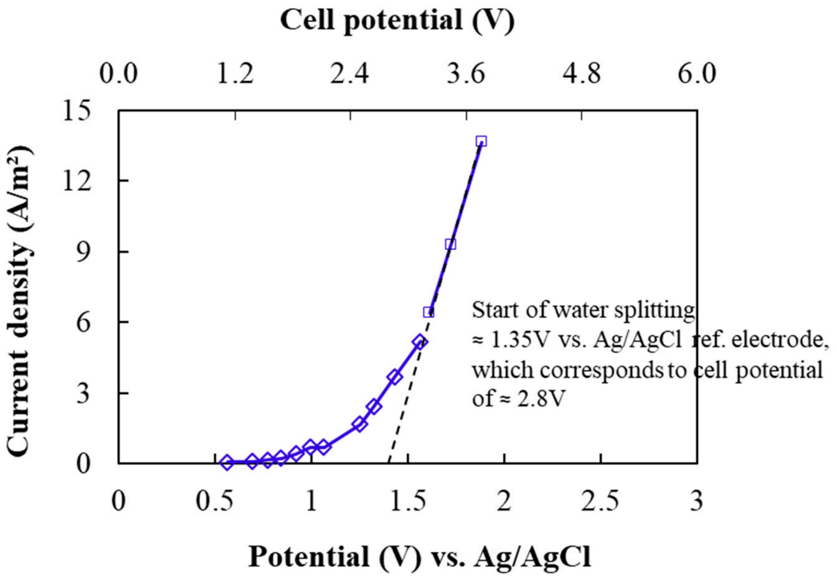

Figure 2S. V-I curve for cell potential in feed solution (natural organic water= 12 $mg \cdot L^{-1}$ with NaCl= 1 $mmol \cdot L^{-1}$ at pH 7 and filtration flux= 100 $L \cdot m^{-2} \cdot h^{-1}$ [2].

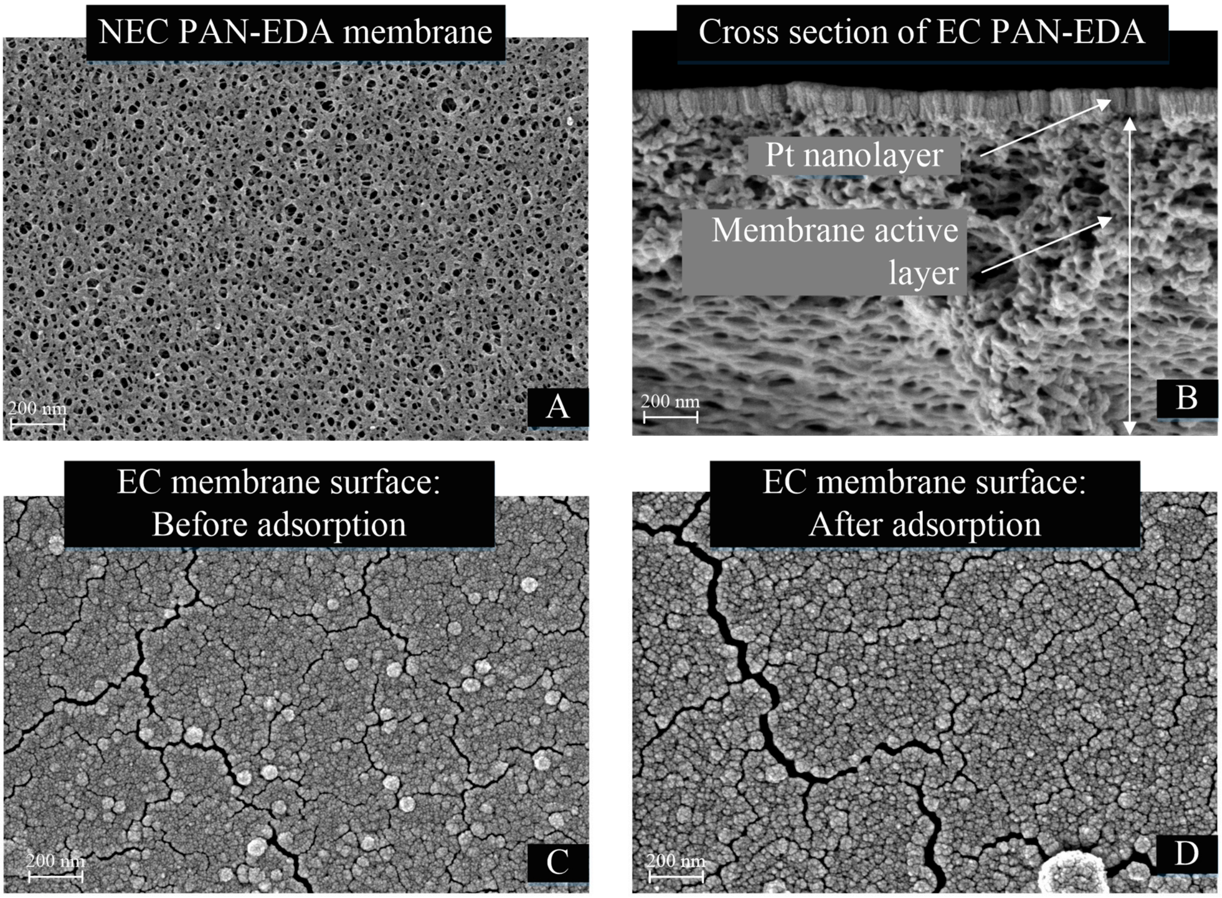

Figure 3S. SEM imaging of (A) uncoated PAN-EDA membrane; (B) cross-section of EC PAN-EDA membrane; (C) EC PAN-EDA membrane: before electro-adsorption; (D) EC PAN-EDA membrane: after electro-adsorption with 50K magnification.

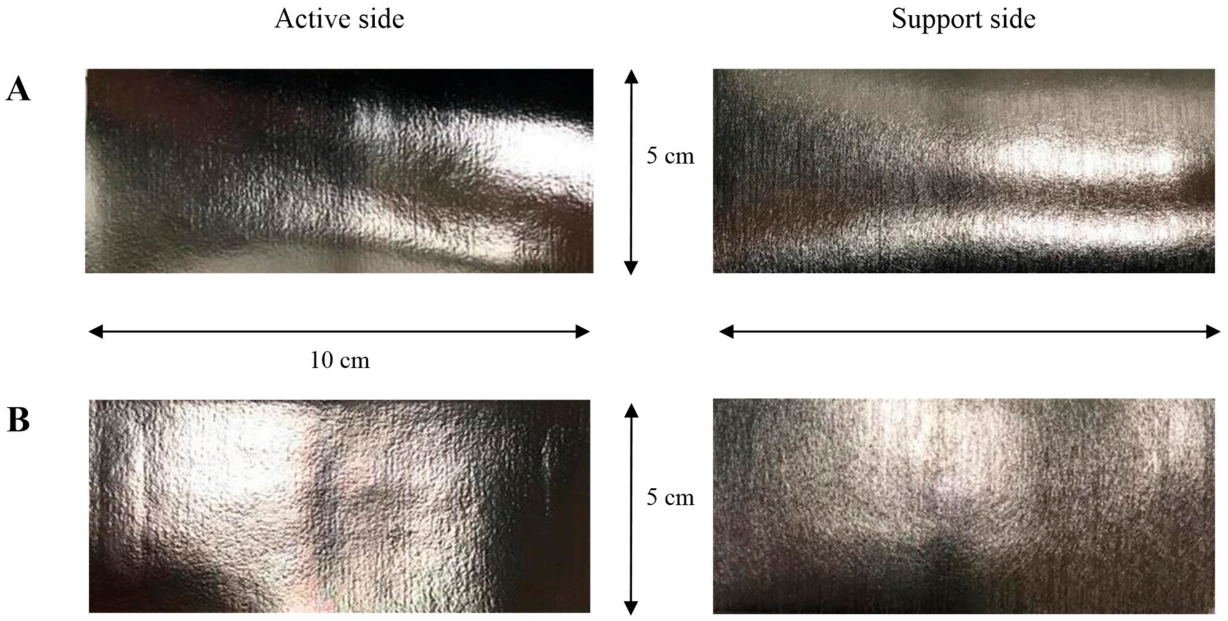

Figure 4S. Visual appearances of the EC membrane; (A) Before electro-adsorption; and B) After membrane regeneration.

References

1. Liu, Y.; Zeng, Y.; Liu, R.; Wu, H.; Wang, G.; Cao, D. Poisoning of acetone to Pt and Au electrodes for electrooxidation of 2-propanol in alkaline medium. *Electrochimica Acta* **2012**, *76*, 174–178, doi:10.1016/j.electacta.2012.04.130.
2. Usman, M.; Glass, S.; Mantel, T.; Filiz, V.; Ernst, M. Electro-sorption and -desorption characteristics of electrically conductive polyacrylonitrile membranes to remove aqueous natural organic matter in dead-end ultrafiltration system. *Journal of Water Process Engineering* **2024**, *58*, 104733, doi:10.1016/j.jwpe.2023.104733.
